# rESWT in Shoulder Periarthritis: Does the Protocol Intensity Matter?—A Quasi-Experimental Non-Randomized Comparative Study

**DOI:** 10.3390/life15060922

**Published:** 2025-06-06

**Authors:** Diana-Lidia Tache-Codreanu, Iuliana David, Ana-Maria Tache-Codreanu, Corina Sporea, Claudia-Camelia Burcea, Dan Corneliu Blendea, Maria-Veronica Morcov, Ioana Elena Cioca

**Affiliations:** 1 Medical Rehabilitation Department, Colentina Clinical Hospital, Stefan cel Mare Street No. 19–21, 020125 Bucharest, Romania; dianatache@yahoo.com (D.-L.T.-C.); julexim@gmail.com (I.D.); 2 Faculty of Midwifery and Nursing, University of Medicine and Pharmacy “Carol Davila”, 37 Dionisie Lupu Street, 020021 Bucharest, Romania; ana-maria.tache2024@stud.umfcd.ro (A.-M.T.-C.); claudia_burcea@yahoo.com (C.-C.B.); ioana.cioca@umfcd.ro (I.E.C.); 3 National University Center for Children Neurorehabilitation “Dr. Nicolae Robanescu”, 44 Dumitru Minca Street, 041408 Bucharest, Romania; 4 Faculty of Medicine, “Titu Maiorescu” University of Bucharest, 031593 Bucharest, Romania; danblendea@gmail.com

**Keywords:** shoulder periarthritis, extracorporeal shockwave therapy, rehabilitation, tendon regeneration

## Abstract

Radial extracorporeal shockwave therapy (rESWT) is used in the rehabilitation of patients with shoulder periarthritis (SP) to promote tendon regeneration. This quasi-experimental non-randomized comparative study included 36 cases of SP, divided into two groups, and aimed to comparatively investigate the analgesic and functional effects of two different rESWT protocols. In Group One, the protocol involved an energy level of 210 J/session, a frequency of 10 Hz, and 2500 impulses per session. In Group Two, the protocol used an energy level of 190 J/session, a frequency of 10–15 Hz, and 2000 impulses per session. Treatments were administered over three sessions in Group One and five sessions in Group Two, with one-week intervals between sessions. Both rESWT protocols were combined with a physical therapy program consisting of ten daily sessions of analgesic physiotherapy and kinesiotherapy. Before and after the rehabilitation program, patients were assessed for pain intensity using the visual analog scale (VAS) and shoulder function using range of motion (ROM) measurements (via goniometry) and the Shoulder Pain and Disability Index (SPADI). Significantly better outcomes were observed in Group One (*p* < 0.001), particularly in terms of pain reduction and improvements in shoulder functionality, especially external rotation. These results support the effectiveness of the rESWT protocol used in Group One, which combined lower energy and frequency levels with a higher number of impulses over fewer sessions.

## 1. Introduction:

Shockwaves are single, short-lived sonic pulses (lasting approximately 10 microseconds) with high peak pressures (up to 500 bar) that rise in less than 10 nanoseconds and are delivered to a specific area [1], typically at frequencies between 16 Hz and 20 Hz [2]. Extracorporeal shockwave therapy (ESWT) was initially introduced in 1980 as a medical intervention for the disintegration of kidney stones [3]. Over the past few decades, its applications have expanded to the treatment of musculoskeletal conditions such as plantar fasciitis, shoulder tendinopathy, Achilles tendinopathy, and myofascial pain syndrome [4] and even to the stimulation of bone growth [5].

ESWT has been shown to induce neovascularization at tendon insertion sites by enhancing blood flow [6,7], to promote cell proliferation and tissue regeneration, and to exert analgesic and anti-inflammatory effects, including nociceptive inhibition [8]. The physical mechanisms involved include stress-related phenomena caused by the rapid rise in pressure (less than 10 nanoseconds) and the formation of cavitation bubbles at solid–liquid interfaces, which may lead to microvascular damage and trigger angiogenesis in soft tissue [6,7,8]. The therapeutic effects of ESWT are dose-dependent, and the “more is better” principle has been suggested in some contexts [2,9,10,11].

Shockwave therapy remains a relatively new, non-invasive therapeutic intervention [4], and no serious adverse events have been widely reported [11], with the exception of a single case report describing humeral head osteonecrosis [12]. Minor side effects—such as pain, local soft tissue swelling, cutaneous erosions, erythema, and subcutaneous hematomas—may occur [13,14], but they typically resolve after the treatment ends.

There are two main types of ESWT: focused (fESWT) and radial (rESWT). fESWT involves sonic pulses generated by electrohydraulic, electromagnetic, or piezoelectric technology, which are converted into focused acoustic pressure waves with the highest energy concentrated at a specific target within the pathological tissue [13]. In contrast, rESWT is generated by a projectile fired through a guiding tube that strikes a metal applicator placed on the skin. This impact generates stress waves that are transmitted as pressure waves into the tissue [13]. To date, there is no conclusive scientific evidence favoring either rESWT or fESWT in terms of treatment outcomes [10,11,15,16].

Periarthritis of the shoulder is a condition characterized by shoulder pain, stiffness, and functional limitations in both active and passive shoulder movements [17,18,19] and may often be associated with calcifying tendinitis [20,21,22,23]. The diagnosis is based on clinical findings such as joint stiffness, severe pain—especially at night—and a significant reduction in shoulder range of motion (ROM) [17,24,25]. The most important clinical sign is the loss of passive external rotation, as radiographic imaging typically appears normal [26].

The key to recovery is restoring motion, but movement is often limited by pain, which contributes to muscle inhibition. The primary purpose of extracorporeal shockwave therapy (ESWT) in this context is to reduce pain, enabling the early initiation of ROM exercises and functional rehabilitation.

Several studies have demonstrated that ESWT is effective in reducing pain and improving shoulder function and also quality of life in patients with calcifying tendinopathy [1,2,3,4,5,27,28,29,30,31,32]. However, evidence regarding its efficacy in shoulder periarthritis, with or without calcific deposits, remains limited. Moreover, it has been shown that pain reduction is not necessarily related to the resorption of calcium deposits [2].

Despite the growing body of research supporting the use of ESWT in shoulder disorders, comparative studies examining different rESWT protocols in terms of the energy levels, frequency, and number of impulses are scarce. Most available studies focus either on single treatment protocols [33,34,35] or on comparisons between ESWT and other conservative therapies, such as physical therapy [36,37], corticosteroid injections [38], hyaluronic acid injections [39], or ultrasound therapy [40,41]. There is a lack of standardized treatment parameters across studies, making it difficult to establish the most effective rESWT protocol for shoulder periarthritis or calcifying tendinitis. This gap in the literature justifies the need for further investigation, particularly in clinical settings, to optimize protocol selection and improve patient outcomes.

This quasi-experimental, non-randomized comparative study aimed to evaluate pain and functional status in individuals diagnosed with shoulder periarthritis, with or without calcinosis, before and after rESWT intervention. The study also compared two rESWT protocols with different parameters (energy, frequency, and number of impulses) to identify the more effective approach.

## 2. Materials and Methods

### 2.1. Study Design

This quasi-experimental, non-randomized clinical study evaluated and compared the effects of two distinct rESWT protocols in patients with periarthritis of the shoulder. Group allocation was determined by the availability of the rESWT device at the time of each patient’s admission to therapy. No randomization or matching procedures were applied.

### 2.2. Setting

The study was conducted at the Medical Rehabilitation Department, Colentina Clinical Hospital, Bucharest, Romania. The study period extended from 2018 to 2025, during which data were collected from clinical records. All interventions and assessments were performed in a single center, ensuring the consistency of the procedures.

### 2.3. Data Collection

Clinical and functional data were collected before the intervention and one week after the final rESWT session. The following outcome measures were used.

- Pain intensity was assessed using the visual analog scale (VAS), with a 10 cm horizontal line where 0 represents “no pain” and 10 indicates the “worst pain imaginable”. The VAS has demonstrated excellent test–retest reliability (ICC = 0.99) [42,43].
- Shoulder function and disability were measured using the Shoulder Pain and Disability Index (SPADI), a validated self-administered questionnaire composed of 13 items: 5 assessing pain and 8 assessing disability. SPADI scores are expressed as percentages and interpreted as follows: 0–20%—mild shoulder pain and disability; 21–40%—moderate; 41–60%—severe; 61–80%—very severe; 81–100%—extremely severe. The numerical version was used, with total scores expressed as percentages and higher values indicating more severe impairment. Its test–retest reliability is high (ICC = 0.89–0.93) [44,45].
- The range of motion (ROM) was evaluated through the goniometric assessment of the shoulder joint, including flexion, extension, abduction, external rotation, and internal rotation, recorded in degrees. Standardized positioning and measurement techniques were used to ensure consistency [46,47].

These measures allowed the evaluation of pain relief, functional improvement, and joint mobility recovery, both within and between the two groups.

### 2.4. Participants

A total of 36 patients diagnosed with shoulder periarthritis were included and divided into two groups: Group One (n = 16) and Group Two (n = 20). Each group received the same multimodal rehabilitation program, differing only in the energy, frequency, and number of impulses applied during the rESWT sessions. Patients were allocated to treatment groups based on device availability at the time of admission, as each group followed a protocol using a different rESWT device. This pragmatic allocation reflects real-world clinical conditions and was not randomized.

### 2.5. Eligibility Criteria

Patients were eligible for inclusion if they were aged between 18 and 79 years, experienced recurrent episodes of shoulder pain lasting for at least 3 to 6 months, and reported a baseline pain intensity of at least 2 cm on the visual analog scale (VAS). A clinical diagnosis of shoulder periarthritis was required, either with or without calcifying tendinopathy.

Patients were excluded if they presented with any of the following: clinical signs of a partial or complete rotator cuff tear, shoulder joint osteophytes, or a history of previous shoulder surgery (primary or revision procedures). Additional exclusion criteria included the presence of a pacemaker, pregnancy, known coagulation disorders, or current anticoagulant therapy, as well as inflammatory or neoplastic musculoskeletal conditions such as rheumatoid arthritis or bone infections.

Patients with acute bursitis confirmed by ultrasound, those who had received a corticosteroid injection in the affected shoulder within the previous six months [2,48,49], and individuals unable or unwilling to comply with the prescribed movement restrictions were also excluded from the study.

No matching was applied, as this was not a matched cohort study.

### 2.6. Intervention and Exposure

All patients followed the same multimodal conservative rehabilitation program. This included the topical application of non-steroidal anti-inflammatory drugs (NSAIDs) in the form of gels or creams, along with ten daily sessions of physiotherapy using transcutaneous electrical nerve stimulation (TENS). TENS was administered via a symmetrical biphasic waveform at a 100 μs pulse duration and 200 Hz base frequency, with random frequency modulation and constant current mode, aiming to reduce pain perception [50,51,52].

In addition, magnetotherapy was delivered using a condition-specific preset titled “Periarthritis of the shoulder”, applying a variable magnetic field of 54 mT and a permanent field intensity of 230 mT for 30 min per session, following a square waveform pattern.

Laser therapy was also included, using a standardized protocol designed for musculoskeletal conditions, applying low-level laser stimulation to the shoulder area to enhance tissue healing and exert analgesic and anti-inflammatory effects [53]. The protocol involved a dose of 20 J/cm^2^ applied to a 25 cm^2^ area, with a maximum power output of 1800 mW.

All patients also participated in a supervised therapeutic exercise program, consisting primarily of pendulum movements, intended to improve shoulder mobility, restore the functional range of motion, and reduce stiffness. Therapeutic exercise is widely recognized as an essential component of conservative management for shoulder disorders, with proven efficacy in reducing pain, enhancing neuromuscular control, and promoting tissue remodeling [54,55,56].

Two different shockwave therapy devices, each dedicated to the treatment of painful shoulder conditions, were used in this study. Each device was operated based on a manufacturer-recommended therapeutic protocol, with preset energy levels, impulse counts, and frequencies tailored to device-specific parameters. Consequently, patients were allocated to two groups corresponding to the equipment used.

The differences in the treatment protocols between the groups—including the frequency and energy settings—reflected the standard operational presets defined by each manufacturer, rather than a custom-designed comparative protocol. This approach ensured consistency within each treatment arm and allowed a pragmatic evaluation of two clinically established therapeutic protocols for shoulder periarthritis.

Group One was treated using the Storz Medical Duolith SD1 ESWT device, which consists of a control unit, a handpiece with three different applicator heads, and a medical air compressor. The compressor generates pneumatic energy that accelerates a projectile inside the handpiece; the shockwave is then created by the impact of the projectile against the applicator tip, propagating radially into the tissue. Treatment in this group was administered using a 15 mm head applicator and consisted of three weekly sessions. The session protocol included 2500 impulses, applied in two stages: 500 impulses at 1.8 bar and 10 Hz, followed by 2000 impulses at 2.5 bar and 10 Hz. Based on published energy approximations [57,58,59], the estimated total energy delivered per session was approximately 210 joules. 

Group Two received treatment with the BTL-6000 SWT Topline device, which also operates based on pneumatic principles. In this system, compressed air is transferred at high pressure into the applicator, where it accelerates a projectile toward a metallic transmitter, generating a shockwave that propagates into the patient’s tissue. A 15 mm applicator head was used, and each subject underwent three to five weekly sessions, depending on the clinical response. The session protocol included 2000 impulses, structured as follows: 500 impulses at 3.0 bar and 15 Hz, followed by 1500 impulses at 2.5 bar and 10 Hz. Based on estimated energy values [28,60], the energy delivered per session was approximately 190 joules.

A coupling gel was applied prior to treatment in both groups to ensure optimal energy transmission at the skin–applicator interface.

No local anesthesia was used during rESWT application in either group.

### 2.7. Outcome Measures and Data Sources

Data were extracted from standardized patient records and included the following:

- Pain intensity (VAS, 0–10 scale);
- Function/disability (SPADI, numerical format, 0–100%);
- Shoulder ROM—goniometric assessment of flexion, extension, abduction, and internal and external rotation.

The same methods and assessors were used for both groups, ensuring comparability in the assessments.

### 2.8. Bias Control

Several measures were implemented to minimize the risk of bias in this study.

All patients underwent the same standardized physiotherapy protocol, delivered in a consistent manner by trained rehabilitation staff. To ensure measurement reliability, all goniometric assessments of the shoulder range of motion were performed by a single, experienced evaluator, using consistent positioning and techniques across all evaluations.

Subjective data were collected using validated and widely used instruments, including the visual analog scale (VAS) for pain and the Shoulder Pain and Disability Index (SPADI) for functional impairment. The use of these tools helped to reduce the variability associated with patient self-reporting [61].

Furthermore, potential confounding was addressed by excluding patients with comorbidities or clinical conditions that could have interfered with the treatment outcomes or introduced heterogeneity into the sample, such as prior shoulder surgery, rotator cuff tears, or inflammatory joint diseases.

### 2.9. Sample Size Considerations

The sample size (n = 36) was determined by the availability of eligible patients over the study period. No formal sample size calculation was performed, given the exploratory nature of the study.

### 2.10. Handling of Variables

Quantitative outcome variables (VAS, SPADI, ROM angles) were analyzed as continuous variables. No artificial grouping was applied. Pain and disability were also presented categorically for descriptive purposes (e.g., mild/moderate/severe based on standard VAS/SPADI ranges).

### 2.11. Statistical Analysis

All statistical analyses were conducted using Microsoft Office Professional Plus 2024 (Excel) and IBM SPSS Statistics version 26 (IBM Corp., Armonk, NY, USA). Data were checked for completeness and accuracy prior to analysis.

Descriptive statistics—including means and standard deviations for normally distributed variables, and medians with interquartile ranges (IQR) for non-normally distributed ones—were computed for all continuous variables; normality was assessed using the Shapiro–Wilk test, and all variables were analyzed as continuous.

To compare the baseline characteristics and outcome measures between the groups, independent-sample *t*-tests were used for normally distributed variables, while the Mann–Whitney U test was applied for non-normally distributed variables.

Within-group changes (pre- vs. post-treatment) were assessed using paired-sample *t*-tests for normally distributed data and the Wilcoxon signed-rank test for non-normally distributed data [62].

The effect size was calculated for all outcome measures. For variables with a normal distribution, Cohen’s d was used, interpreted according to standard thresholds: small (0.2), medium (0.5), and large (≥0.8) effects [63,64]. For non-normally distributed variables, non-parametric effect sizes were computed. In intra-group analyses (pre- vs. post-intervention within each group), we used the Wilcoxon signed-rank test, and the effect size was calculated as r = Z/√N, where Z is the standardized test statistic and N is the sample size. In inter-group comparisons (between-group differences in outcome changes), we used the Mann–Whitney U test, and the effect size was calculated using the formula d = √(Z^2^/(N − 1)), where Z is the standardized test statistic and N the total number of participants. All effect sizes, including those from non-parametric tests, are reported to provide a standardized interpretation of the clinical relevance.

To explore associations between continuous variables, Pearson correlation coefficients (r) were computed separately for each group. Correlation matrices were generated and are provided in the Results section.

No missing data were identified in the dataset. All patients completed the rehabilitation protocol and follow-up evaluations; thus, no imputation or adjustment for missing data was necessary. No sub-group or interaction analyses were pre-planned beyond the comparison of the two protocol groups.

A two-tailed significance level of *p* < 0.05 was used for all tests.

The study did not employ multifactorial statistical models such as ANOVA with multiple factors, as each group received a fixed, manufacturer-defined protocol specific to the device used. Parameters such as the energy level, frequency, and number of impulses were pre-defined and applied uniformly within each group, without within-group variation. Therefore, the inter-group comparisons reflect the clinical effectiveness of complete, standardized rESWT protocols rather than the isolated influences of individual stimulation parameters.

### 2.12. Ethical Considerations

All participants were informed about the potential risks associated with the procedure. Written informed consent was obtained from all subjects. The study protocol was approved by the Research Ethics Committee of Colentina Clinical Hospital, in accordance with the Declaration of Helsinki (approval no. 16/27 July 2017). All data were used exclusively for research purposes and anonymized during analysis.

## 3. Results

### 3.1. Participants

**Figure 1:**
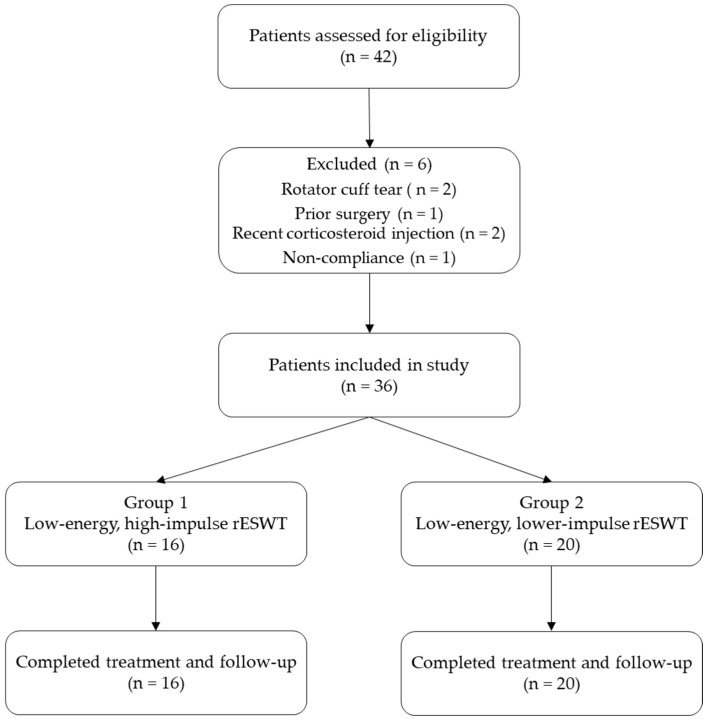
Patients were assessed for eligibility; 36 met the inclusion criteria and were allocated to one of two rESWT protocols.

A total of 42 patients with shoulder periarthritis were initially screened. Of these, 36 met the eligibility criteria and were included in the analysis (Figure 1).

- Group 1: 16 patients received low-energy, high-impulse rESWT.
- Group 2: 20 patients received high-energy, lower-impulse rESWT.

A total of six patients were excluded during the eligibility assessment process for the following reasons:

- Two patients presented with a rotator cuff tear confirmed by ultrasound examination;
- One patient had a history of shoulder surgery;
- Two patients had received a corticosteroid injection in the affected shoulder within the previous six months;
- One patient was excluded due to non-compliance with the prescribed home exercise protocol.

### 3.2. Descriptive Data

The main demographic and clinical characteristics of the participants in both groups are presented in Table 1.

The normality of the data distribution was assessed using the Shapiro–Wilk test, given the relatively small sample size. Only three variables met the criteria for a normal distribution (*p* > 0.05), namely VAS initial, VAS final, and SPADI final. Consequently, descriptive statistics are presented as means ± standard deviations for normally distributed variables and as medians and interquartile ranges (IQRs) for those with a non-normal distribution.

The baseline demographic and clinical characteristics of the study participants are summarized in Table 1. The two groups were compared in terms of age, pain intensity (VAS), functional status (SPADI), and shoulder range of motion (flexion, extension, abduction, internal and external rotation) at baseline. At the beginning of the study, significant differences were observed in the VAS scores (*p* = 0.004), SPADI scores (*p* = 0.010), and most range of motion parameters, with Group 2 demonstrating better initial mobility. The only parameters with normally distributed values in both groups were VAS_initial and Internal_Rotation_initial, for which effect sizes were computed using Cohen’s d. Although the abduction parameter was normally distributed in Group 2, normality was not demonstrated in Group 1; thus, non-parametric tests were applied. Accordingly, Mann–Whitney U tests were used for between-group comparisons of non-normally distributed outcomes, and non-parametric effect sizes were calculated using the formula d = √(Z^2^/(N − 1)), where Z is the standardized test statistic and N the total number of participants.

Values are presented as the mean ± SD or median (IQR), as appropriate. *p*-values were calculated using independent-sample *t*-tests or Mann–Whitney U tests, depending on the data distribution. For all comparisons, effect sizes—either Cohen’s d or non-parametric equivalents—are reported to enhance the interpretation of the clinical relevance.

At baseline, Group 1 exhibited significantly higher levels of pain and functional impairment compared to Group 2. The VAS scores were higher in Group 1 (7.56 ± 1.71) than in Group 2 (5.50 ± 2.14), with a statistically significant difference (*p* = 0.004) and a large effect size (Cohen’s d = 1.05), indicating a clinically relevant disparity in pain perception. Functional disability, measured by the SPADI, was also more severe in Group 1 (median = 74.65, IQR = 68.88–93.10) compared to Group 2 (median = 66.15, IQR = 27.10–75.80), with a statistically significant difference (*p* = 0.010) and a non-parametric effect size of r = 0.44, suggesting a moderate-to-large magnitude of difference.

Non-parametric comparisons further revealed that Group 2 had a significantly greater initial range of motion in shoulder flexion (*p* = 0.031, r = 0.36), abduction (*p* = 0.006, r = 0.47), and external rotation (*p* = 0.002, r = 0.30). No significant differences were found in age (*p* = 0.388), extension (*p* = 0.334), or internal rotation (*p* = 0.953), with the latter also showing a negligible effect size (Cohen’s d = 0.02).

There were no missing data for any variable included in the analysis.

Each patient was followed up with for the duration of the rehabilitation program, with a final assessment one week after the last rESWT session. As rESWT was applied weekly for three or five sessions (depending on the group), the total follow-up time ranged from 4 to 6 weeks. No adverse effects or complications related to the intervention were reported in either group throughout the study period.

### 3.3. Outcome Data

All outcome measures were collected before treatment (baseline) and after completion of the rESWT protocol. These included the following:

- Pain intensity (VAS);
- Function/disability (SPADI);
- Shoulder ROM—flexion, extension, abduction, and external and internal rotation.

**Figure 2:**
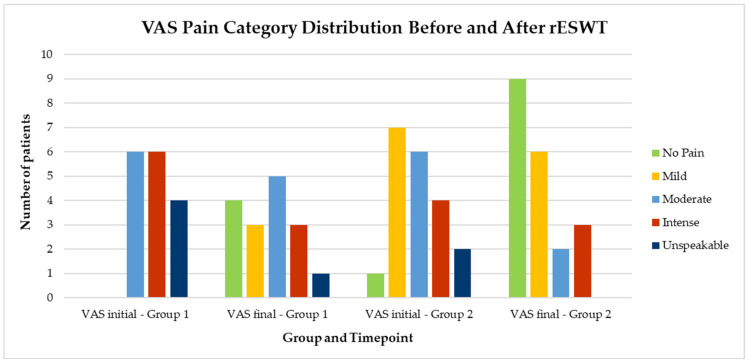
Distribution of patients by VAS pain intensity category before and after rESWT treatment in both groups. Note. VAS categories: No pain (0), mild (1–3), moderate (4–6), intense (7–8), unspeakable (9–10). rESWT = radial extracorporeal shockwave therapy. The bars indicate the number of patients in each pain category at baseline and post-treatment in Group 1 and Group 2.

**Figure 3:**
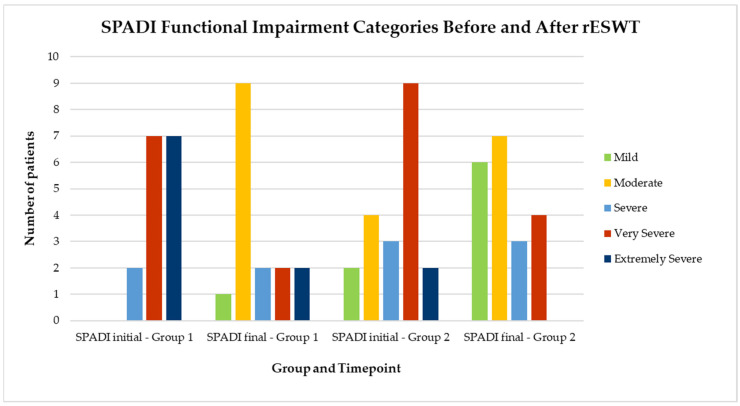
Distribution of patients by SPADI functional impairment category before and after rESWT treatment in both groups. Note. SPADI categories: mild (0–20%), moderate (21–40%), severe (41–60%), very severe (61–80%), extremely severe (81–100%). The bars indicate the number of patients in each functional category before and after treatment with radial extracorporeal shockwave therapy (rESWT), in Group 1 and Group 2.

**Figure 4:**
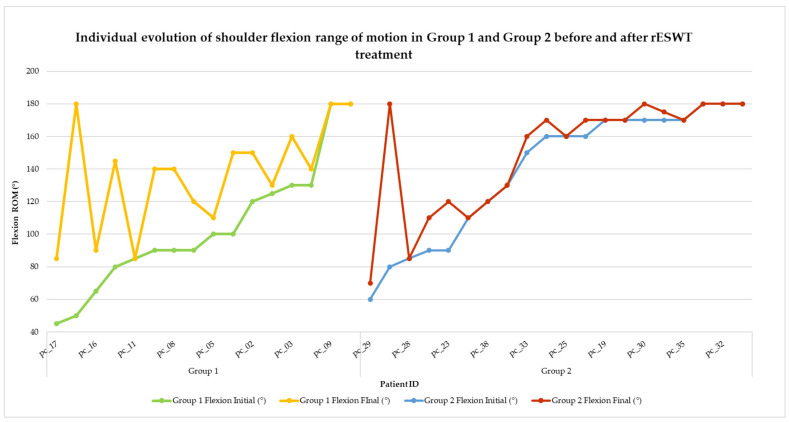
Individual evolution of shoulder flexion range of motion in Group 1 and Group 2 before and after rESWT treatment. Note. This figure displays the ROM progression for each patient. Group 1 is represented by green (initial) and yellow (final) lines, while Group 2 is represented by blue (initial) and red (final) lines. A consistent increase in flexion was observed in both groups, with Group 1 generally demonstrating a greater overall improvement.

**Figure 5:**
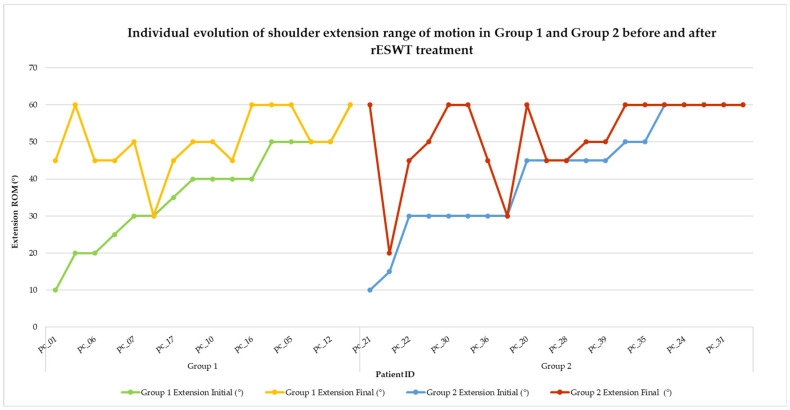
Individual evolution of shoulder extension range of motion in Group 1 and Group 2 before and after rESWT treatment. Note. Each line represents one patient’s shoulder extension ROM before and after rESWT. Group 1 is illustrated by green (initial) and yellow (final) lines; Group 2 is represented by blue (initial) and red (final) lines. Improvements are visible in both groups, with Group 1 showing a more consistent upward trend.

**Figure 6:**
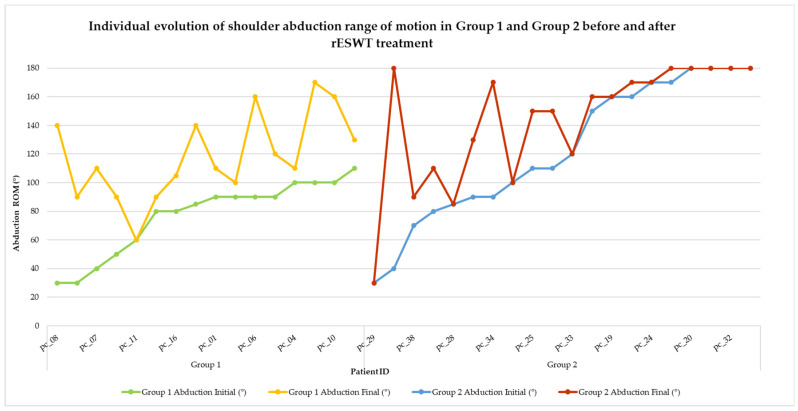
Individual evolution of shoulder abduction range of motion in Group 1 and Group 2 before and after rESWT treatment. Note. Each line represents one patient’s shoulder abduction ROM before and after treatment. Group 1 is illustrated by green (initial) and yellow (final) lines, while Group 2 is represented by blue (initial) and red (final) lines. A general upward trend is observed in both groups, with Group 1 demonstrating a more consistent and pronounced improvement.

**Figure 7:**
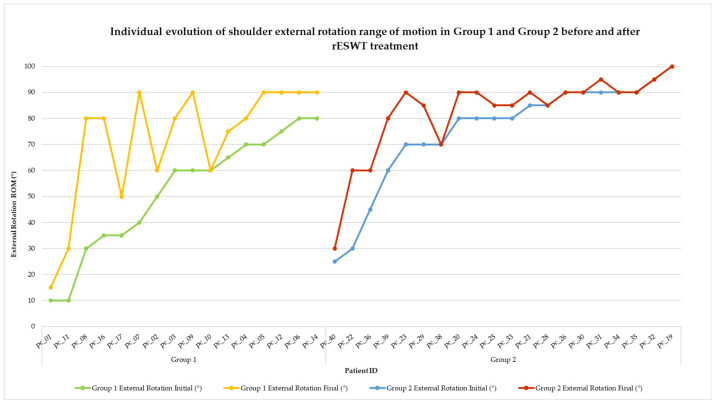
Individual evolution of shoulder external rotation range of motion in Group 1 and Group 2 before and after rESWT treatment. Note. Each line shows the external rotation ROM for one patient before and after rESWT. Group 1 is represented by green (initial) and yellow (final) lines and Group 2 by blue (initial) and red (final) lines. While both groups improved, Group 1 exhibited more substantial gains across a broader patient range.

**Figure 8:**
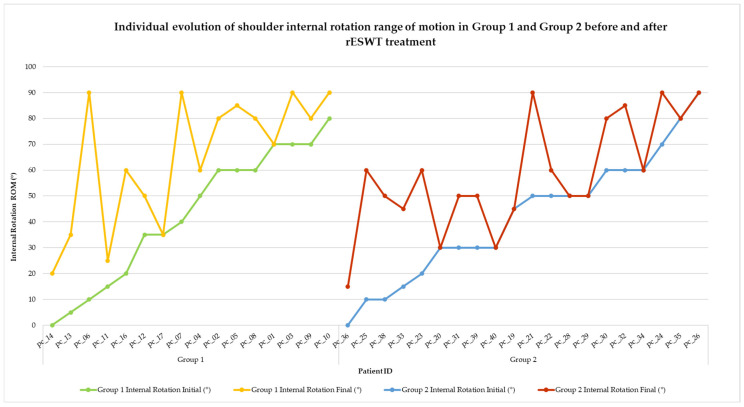
Individual evolution of shoulder internal rotation range of motion in Group 1 and Group 2 before and after rESWT treatment. Note. Each line represents the internal rotation ROM for a single patient before and after rESWT. Group 1 is represented by green (initial) and yellow (final) lines and Group 2 by blue (initial) and red (final) lines. Improvements were observed in both groups, with Group 1 showing greater amplitude gains and fewer post-treatment plateaus.

Visual representations of categorical changes in pain (VAS) and function (SPADI) are shown in Figure 2 and Figure 3, while individual progressions in range of motion (ROM) are illustrated in Figure 4, Figure 5, Figure 6, Figure 7 and Figure 8.

To illustrate the clinical evolution of pain perception, patients’ VAS scores were categorized into five severity levels and compared pre- and post-treatment. Figure 2 shows the distribution of patients across these pain intensity categories in both study groups. A visible shift toward lower pain categories is observed post-treatment, especially in Group 2, where most patients reported mild or no pain following the intervention.

To further illustrate the impact of rESWT on shoulder function and pain, patients were categorized according to their Shoulder Pain and Disability Index (SPADI) scores. Based on the percentage values, the SPADI scores were classified as follows: mild (0–20%), moderate (21–40%), severe (41–60%), very severe (61–80%), and extremely severe (81–100%). Figure 3 shows the distribution of patients across these SPADI severity categories, before and after treatment, in both groups. A visible shift toward lower disability levels is observed in both groups, especially in Group 1, where most patients moved from the “very severe” and “extremely severe” categories to “moderate” or “mild” levels after the intervention.

To evaluate the progression of shoulder mobility following rESWT, the shoulder flexion range of motion (ROM) was recorded for each patient before and after treatment. The results are illustrated in Figure 4, which displays the individual trajectory of improvement in both groups.

Shoulder extension is essential for posterior capsule flexibility and contributes to numerous functional movements, such as reaching behind the body. The range of motion (ROM) in extension was evaluated pre- and post-treatment in both groups. The individual progression of shoulder extension is shown in Figure 5, illustrating treatment-related gains for each patient.

Shoulder abduction is a key component of shoulder mobility, particularly relevant for overhead activities and daily functions such as dressing or reaching. To assess the impact of rESWT on this movement, the range of motion (ROM) in shoulder abduction was recorded before and after treatment for each patient. The individual results for both groups are illustrated in Figure 6, showing the pre- and post-treatment trajectories.

The external rotation of the shoulder is crucial for everyday functions such as grooming, dressing, or reaching behind the head. This movement is frequently restricted in patients with shoulder periarthritis. Figure 7 presents the individual evolution of the external rotation range of motion (ROM) before and after treatment with rESWT in both groups.

The internal rotation of the shoulder is essential for daily activities such as fastening garments behind the back or reaching into a back pocket. In patients with shoulder periarthritis, this motion is often significantly limited. Figure 8 illustrates the individual evolution of the internal rotation ROM in both treatment groups, before and after rESWT.

### 3.4. Main Results

Table 2 presents the intra-group comparisons of the clinical outcomes assessed before and after the intervention, separately for Group 1 and Group 2. Changes in pain intensity (VAS), functional status (SPADI), and shoulder range of motion (ROM) were evaluated to quantify the treatment effects over time. Depending on the data distribution, either paired-sample *t*-tests (for normally distributed variables) or Wilcoxon signed-rank tests (for non-normally distributed variables) were applied. The results are reported as the mean ± standard deviation or median (interquartile range), accompanied by *p*-values and effect sizes. For normally distributed variables, effect sizes were calculated using Cohen’s d; for non-normally distributed variables, non-parametric effect sizes were computed using the formula r = Z/√N, where Z is the standardized test statistic and N is the sample size. These values support the interpretation of both statistical significance and clinical relevance.

Table 3 presents the between-group comparisons of the change scores (Δ), computed as the difference between the post-treatment and baseline values for each clinical outcome measure. These comparisons were used to evaluate whether the two radial extracorporeal shockwave therapy (rESWT) protocols resulted in significantly different clinical improvements. Depending on the data distribution, either independent-sample *t*-tests or Mann–Whitney U tests were applied. Effect sizes were calculated for all comparisons: Cohen’s d was used for normally distributed variables, while, for non-normally distributed variables, a non-parametric effect size was computed using the formula d = √(Z^2^/(N − 1)), where Z is the standardized test statistic from the Mann–Whitney U test and N is the total number of participants.

The within-group comparisons using paired *t*-tests confirmed the statistically significant improvements across all outcomes in both groups.

### 3.5. Absolute Risk Translation

Not applicable, as this was not a risk-based or prospective study.

### 3.6. Other Analyses

To explore the associations between the baseline characteristics and treatment outcomes, correlation analyses were performed separately for each group.

Pain at baseline, measured by VAS initial, showed strong and significant correlations with both the post-treatment pain levels (r = 0.602, *p* = 0.014) and initial SPADI scores (r = 0.625, *p* = 0.010), reflecting the known interplay between perceived pain and functional limitations.

An improvement in pain (VAS difference) was closely associated with functional improvements, with a strong correlation between pain reduction and SPADI improvements (r = 0.863, *p* < 0.001).

Motor recovery appeared synergistic: flexion gains were moderately correlated with internal rotation gains (r = 0.608, *p* = 0.012), while the final flexion correlated strongly with the final abduction (r = 0.756, *p* = 0.001) and final internal rotation (r = 0.658, *p* = 0.006), suggesting a coordinated recovery pattern.

Additionally, the final external rotation correlated with internal rotation gains (r = 0.524, *p* = 0.037).

In Group 2, the initial VAS had even stronger correlations with both the final VAS (r = 0.762, *p* < 0.001) and initial SPADI (r = 0.802, *p* < 0.001), emphasizing pain’s predictive role in perceived disability.

Regarding ROM, the final internal rotation was strongly associated with the final external rotation (r = 0.498, *p* = 0.025), suggesting coordinated motor recovery across planes.

To provide a comprehensive overview of the relationships between the clinical and functional variables, the full Pearson correlation matrices for each group are presented in Table 4 (Group 1) and Table 5 (Group 2). These tables include all pairwise correlations among the baseline measures, post-treatment outcomes, and ROM gains.

## 4. Discussion

This study explored the clinical efficacy of two distinct rESWT protocols in patients with periarthritis of the shoulder (PSH), a condition that continues to challenge rehabilitation professionals due to its complex pathophysiology and variable response to treatment. Both treatment groups showed statistically significant improvements in pain (VAS), function (SPADI), and range of motion (ROM), confirming that rESWT represents a promising adjunct to multimodal rehabilitation in PSH [65,66]. However, superior gains in shoulder flexion and external rotation were observed in the group treated with the lower-energy, higher-impulse protocol, highlighting the potential impact of specific energy dosing patterns on the clinical outcomes.

The baseline comparisons revealed several statistically and clinically relevant differences between the two groups. Group 1 reported significantly higher pain levels at baseline compared to Group 2 (VAS: 7.56 ± 1.71 vs. 5.50 ± 2.14; *p* = 0.004, Cohen’s d = 1.05), indicating a large effect size. Functional disability, assessed using the SPADI index, was also more severe in Group 1 (median = 78.38, IQR not applicable due to mean presentation) than in Group 2 (median = 66.15, IQR = 27.10–75.80; *p* = 0.010; r = 0.436), reflecting a moderate to large effect.

Moreover, the initial range of motion was significantly more limited in Group 1 in the following key directions:

- Flexion—103.75° vs. 160.00°, *p* = 0.031, r = 0.365;
- Abduction—87.50° vs. 122.75°, *p* = 0.006, r = 0.468;
- External rotation—51.88° vs. 80.00°, *p* = 0.002, r = 0.520.

No significant differences were found between the groups in terms of age (*p* = 0.388, r = 0.146), extension (*p* = 0.334, r = 0.163), or internal rotation (*p* = 0.953, Cohen’s d = 0.02), suggesting comparable baseline values for these parameters.

The intra-group analyses demonstrated statistically significant improvements in both groups following rESWT, across all clinical outcomes. In Group 1, the pain levels (VAS) decreased markedly (*p* < 0.001, Cohen’s d = 1.31), accompanied by substantial gains in functional status (SPADI, *p* < 0.001, r = 0.622) and range of motion, including flexion (*p* = 0.001, Cohen’s d = 0.918), abduction (*p* = 0.001, r = 0.604), and external rotation (*p* = 0.001, r = 0.606). The effect sizes ranged from moderate to large for all parameters, including extension (*p* = 0.002, r = 0.546) and internal rotation (*p* = 0.001, r = 0.585).

Similarly, Group 2 exhibited statistically significant post-treatment gains in all assessed domains. Pain reduction reached significance (*p* < 0.001, Cohen’s d = 0.988), as did functional improvement (SPADI, *p* < 0.001, r = 0.620) and increases in internal rotation (*p* < 0.001, Cohen’s d = 0.715). The range of motion improvements were also significant, albeit more modest than those observed in Group 1, with effect sizes in the moderate range for flexion (*p* = 0.007, r = 0.486), extension (*p* = 0.002, r = 0.446), abduction (*p* = 0.005, r = 0.488), and external rotation (*p* = 0.002, r = 0.429).

These results confirm the clinical efficacy of rESWT within each treatment group. However, the larger functional gains in Group 1—despite receiving fewer impulses—support the hypothesis that the intensity and distribution of shockwaves may have a greater therapeutic impact than the total energy delivered. This finding aligns with the existing literature suggesting that the treatment parameters should be optimized based on the physiological response rather than the total dosage alone.

The inter-group comparisons revealed statistically significant differences favoring Group 1 for key functional outcomes, particularly in shoulder flexion (*p* = 0.005, r = 0.477), abduction (*p* = 0.011, r = 0.430), and external rotation (*p* = 0.004, r = 0.492). Although the SPADI improvement did not reach conventional significance (*p* = 0.072), the effect size was moderate (r = 0.304), suggesting a clinically meaningful trend.

In contrast, the differences in extension (*p* = 0.328, r = 0.165) and internal rotation (*p* = 0.390, r = 0.145) were not statistically significant, and pain relief (VAS) showed comparable reductions in both groups (*p* = 0.153, Cohen’s d = 0.345).

These findings reinforce the superior functional benefits associated with the lower-energy, higher-pressure protocol used in Group 1. However, the observed between-group differences should be interpreted with caution due to baseline imbalances. Group 1 had significantly worse initial scores in several domains, potentially creating greater room for improvement. Notably, no significant inter-group differences emerged for outcome variables that were similar at baseline, suggesting that baseline disparities may have partially driven the observed effects.

Shoulder periarthritis, which includes a spectrum of conditions such as adhesive capsulitis and rotator cuff-related pain, is a prevalent musculoskeletal disorder, affecting up to 20% of adults during their lifetime [67]. The clinical burden of shoulder periarthritis is amplified not only by frequent relapses and functional limitations but also by the psychological distress that they induce. In turn, a poor mental state may hinder treatment adherence and delay clinical improvement, creating a negative feedback loop. Given this bidirectional relationship, healthcare professionals should consider both the mental and physical dimensions of health when evaluating progress and tailoring rehabilitation strategies [68,69,70]. Although corticosteroid injections and physiotherapy remain first-line treatments, recent systematic reviews and meta-analyses suggest that ESWT—especially radial forms—may provide superior or at least comparable outcomes in terms of pain relief and function [71,72,73].

The mechanistic rationale behind rESWT includes the stimulation of neovascularization, the modulation of inflammation, and neuromuscular facilitation. The present findings support these principles and suggest that the treatment protocol matters—not only whether rESWT is applied but how it is delivered, including the pressure, frequency, and number of impulses. Previous studies have reported varied clinical responses depending on these parameters, particularly in tendinopathies and calcific shoulder conditions [74,75,76]. However, comparisons of entire protocols, as performed here, are more aligned with real-world clinical practice than parameter-isolated trials.

Calcific tendinitis remains a frequent comorbidity in PSH, and its presence may influence the therapeutic response [77,78]. While radiologic classification systems have attempted to predict outcomes, current evidence suggests that the pain severity and disability perception are more closely linked to individual response patterns. In our cohort, patients with and without calcification were not separated analytically due to sample size constraints. Nevertheless, both protocols yielded positive outcomes across the board, confirming prior results regarding rESWT in both calcific and non-calcific variants of PSH.

Interestingly, the higher number of total impulses in Group 2 (10,000 vs. 7500) did not result in superior outcomes. In contrast, the lower-energy protocol with fewer impulses appeared more effective for key functional motions, suggesting that the dose intensity may outweigh the total dose volume, as also suggested in the comparative ESWT literature [79]. These results reflect similar patterns found in other rehabilitation contexts, where the total input is not always predictive of functional gains, especially in pediatric neuromotor disorders [80].

Another strength of the current study is the inclusion of a robust correlation analysis. In both groups, the baseline VAS scores were significantly associated with both functional impairment and post-treatment outcomes, highlighting the central role of pain in functional disability [81,82,83]. This relationship between pain, function, and recovery trajectories is consistent with findings from other rehabilitation domains, including post-surgical and respiratory programs, where functional scoring systems have demonstrated similar alignment between pain reduction, range of motion, and the global recovery status [84].

Furthermore, the strong correlations between the flexion, abduction, and internal rotation gains support a synergistic recovery model in shoulder mobility. These findings resonate with earlier motor rehabilitation models in both orthopedic and neurological populations, including studies on post-COVID-19 recovery, pediatric rehabilitation, and robotic-assisted interventions [85,86,87,88].

This study also stands out for its pragmatic approach, in which two complete, manufacturer-recommended treatment protocols were compared under real-world clinical conditions, without artificially isolating or manipulating individual parameters. This type of design reflects the actual way in which therapies are applied in routine rehabilitation settings, aiming to assess their effectiveness rather than the idealized efficacy. This is consistent with previous pragmatic trials in musculoskeletal rehabilitation, such as the UK FROST trial [89], and enhances the external validity of our findings by improving their generalizability to everyday clinical practice. Given this context, a multifactorial ANOVA—which requires strict control over individual variables—was not appropriate for the study design.

### 4.1. Limitations

Our study has several limitations that must be acknowledged when interpreting the results. First, the sample size was moderate, and no stratification was performed based on the calcification status or symptom duration, which might influence the responsiveness to rESWT. Second, the study lacked long-term follow-up, limiting our understanding of the durability of the improvements over time. Third, although both intervention protocols followed the manufacturer recommendations, two different rESWT devices were used. This introduces an element of variability, although it reflects the real-world heterogeneity in clinical settings.

Fourth, no imaging or biochemical outcomes (e.g., inflammatory markers or neovascularization indices) were assessed. Including such parameters, as demonstrated in pediatric and neurodegenerative rehabilitation trials, might provide valuable insights into the biological mechanisms underpinning the observed effects. Fifth, the study did not include a conventional control group (e.g., sham rESWT or rehabilitation-only), which limited our ability to fully isolate the specific contribution of rESWT to the clinical improvements.

An additional and important limitation is the presence of significant baseline differences between the groups, particularly with Group 1 showing higher initial pain and disability levels and a more restricted range of motion. These baseline differences—especially the worse clinical status of Group 1—must be carefully considered when interpreting the post-treatment outcomes. Because the study design was non-randomized, the improvements observed in Group 1 may have been partially driven by regression to the mean or by a greater capacity for clinical gain. This limitation underscores the need for caution when comparing outcome changes between groups and highlights the importance of baseline equivalence in intervention studies.

Finally, while this study primarily focused on pain and joint mobility outcomes, full functional restoration remains the ultimate rehabilitation goal. Future research could benefit from integrating performance-based assessments or wearable sensors to contextualize the improvements, as demonstrated in other musculoskeletal and neurological rehabilitation settings.

### 4.2. Future Research Directions

Given the preliminary nature of this study and the relatively small sample size, further research is warranted to validate and expand upon these findings. Future studies should aim to include larger and more diverse populations to enhance the generalizability. Extending the duration of the intervention and follow-up periods would also be beneficial in assessing the long-term effectiveness and sustainability of the observed improvements. Moreover, incorporating objective biomechanical or imaging assessments could complement patient-reported and functional outcomes, providing a more comprehensive understanding of the mechanisms behind treatment efficacy. Finally, comparative studies including other physiotherapeutic approaches or multidisciplinary strategies may help to delineate the most effective protocols for the management of adhesive capsulitis in different patient sub-groups.

In light of the increasing clinical interest in optimizing non-invasive interventions for shoulder disorders, continued research integrating clinical, imaging, and biomechanical perspectives remains essential to guide evidence-based rehabilitation practice.

## 5. Conclusions

The rehabilitation program applied to Group 1 led to significant improvements in pain levels, shoulder mobility, and functional ability. Several key conclusions can be drawn.

- Pain relief, as measured by the VAS scale, was strongly associated with functional improvements (SPADI), reinforcing the critical role of effective pain management in the success of rehabilitation protocols.
- The recovery of shoulder mobility—particularly in flexion, abduction, and internal rotation—was closely linked to improved functional performance, highlighting the importance of incorporating movement-specific goals into therapy plans.

Overall, these findings emphasize the importance of personalized, multi-modal rehabilitation programs that address both objective clinical parameters and subjective patient experiences. Their integration into routine clinical practice may enhance the therapeutic outcomes in individuals with shoulder dysfunction.

## Figures and Tables

**Table 1:** Baseline clinical comparison between groups.

Parameter	Group 1 (n = 16)	Group 2 (n = 20)	*p*-Value	Effect Size (r)
Age (years)	64.00 (59.25 to 65.00)	64.00 (47.00 to 73.00)	0.388	0.146
VAS Initial	7.56 ± 1.71	5.50 ± 2.14	0.004	1.051 (Cohen’s d)
SPADI Initial	78.38 ± 15.33	66.15 (27.10 to 75.80)	0.010	0.436
Flexion Initial (°)	103.75 ± 39.22	160.00 (95.00 to 170.00)	0.031	0.365
Extension Initial (°)	36.88 ± 13.65	45.00 (30.00 to 57.50)	0.334	0.163
Abduction Initial (°)	87.50 (52.50 to 97.50)	122.75 ± 48.98	0.006	0.468
External Rotation Initial (°)	51.88 ± 22.87	80.00 (70.00 to 90.00)	0.002	0.303
Internal Rotation Initial (°)	42.50 ± 26.20	42.00 ± 24.46	0.953	0.02 (Cohen’s d)

Note. Values are reported as mean ± standard deviation (SD) for normally distributed variables and as median (interquartile range, IQR) for variables with non-normal distribution. Statistical comparisons between groups were performed using independent-sample *t*-tests for normally distributed variables and Mann–Whitney U tests for non-normally distributed variables. Effect sizes were calculated for all comparisons: Cohen’s d was used for parametric tests (independent-sample *t*-tests), and non-parametric effect size r was computed for Mann–Whitney U tests using the formula r = Z/√(N − 1), where Z is the standardized test statistic and N the total number of participants. VAS = visual analog scale; SPADI = Shoulder Pain and Disability Index.

**Table 2:** Intra-group comparisons for clinical outcomes (initial vs. final values).

Group	Variable	Baseline (Mean ± SD/Median, IQR)	Final (Mean ± SD/Median, IQR)	Difference (Mean ± SD/Median, IQR)	*p*-Value	Effect Size (r)
Group 1						
	VAS	7.56 ± 1.71	4.69 ± 2.58	−2.87 ± 2.06	<0.001	1.31 (Cohen’s d)
	SPADI	78.38 ± 15.33	33.10 (25.00 to 70.98)	−31.90 (−46.90 to 16.17)	<0.001	0.622
	Flexion (°)	103.75 ± 39.22	136.56 ± 31.82	32.81 ± 33.21	0.001	0.918 (Cohen’s d)
	Extension (°)	36.88 ± 13.65	50.00 (45.00 to 60.00)	10.00 (1.25 to 20.00)	0.002	0.546
	Abduction (°)	87.50 (52.50 to 97.50)	117.81 ± 30.28	35.00 (12.50 to 67.50)	0.001	0.604
	External Rotation (°)	51.88 ± 22.87	80.00 (60.00 to 90.00)	15.00 (10.00 to 27.50)	0.001	0.606
	Internal Rotation (°)	42.50 ± 26.20	75.00 (38.75 to 88.75)	20.00 (10.00 to 28.75)	0.001	0.585
Group 2						
	VAS	5.50 ± 2.14	3.25 ± 2.40	−2.25 ± 1.59	<0.001	0.988 (Cohen’s d)
	SPADI	66.15 (27.10 to 75.80)	31.85 ± 22.77	−20.62 (−36.92 to −13.05)	<0.001	0.620
	Flexion (°)	160.00 (95.00 to 170.00)	170 (120.00 to 178.75)	0.00 (0.00 to 10.00)	0.007	0.486
	Extension (°)	45.00 (30.00 to 57.50)	60.00 (45.00 to 60.00)	5.00 (0.00 to 15.00)	0.002	0.446
	Abduction (°)	122.75 ± 48.98	160.00 (112.50 to 180.00)	5.00 (0.00 to 37.50)	0.005	0.488
	External Rotation (°)	80.00 (70.00 to 90.00)	90.00 (81.25 to 90.00)	5.00 (0.00 to 13.75)	0.002	0.429
	Internal Rotation (°)	42.00 ± 24.46	58.50 ± 21.59	16.50 ± 16.71	<0.001	0.715 (Cohen’s d)

Note. Results are reported as mean ± standard deviation or median (interquartile range), depending on data distribution. *p*-values < 0.05 were considered statistically significant. For normally distributed variables, effect sizes were calculated using Cohen’s d and interpreted using conventional thresholds: small (0.2), medium (0.5), and large (≥0.8). For non-normally distributed variables, non-parametric effect sizes were computed using the formula r = Z/√N, where Z is the standardized test statistic from the Wilcoxon signed-rank test and N is the sample size. These effect sizes provide a standardized estimate of the magnitude of change within each group.

**Table 3:** Inter-group comparisons for clinical outcomes.

Variable	Δ Group 1 (Mean ± SD/Median, IQR)	Group 2 (Mean ± SD/Median, IQR)	*p*-Value	Effect Size (r)
VAS	−2.87 ± 2.06	−2.25 ± 1.59	0.153	0.345 (Cohen’s d)
SPADI	−31.90 (−46.90 to 16.17)	−20.62 (−36.92 to −13.05)	0.072	0.304
Flexion (°)	32.81 ± 33.21	0.00 (0.00 to 10.00)	0.005	0.476
Extension (°)	10.00 (1.25 to 20.00)	5.00 (0.00 to 15.00)	0.328	0.165
Abduction (°)	35.00 (12.50 to 67.50)	5.00 (0.00 to 37.50)	0.011	0.430
External Rotation (°)	15.00 (10.00 to 27.50)	5.00 (0.00 to 13.75)	0.004	0.492
Internal Rotation (°)	20.00 (10.00 to 28.75)	16.50 ± 16.71	0.39	0.145

Note. Values are reported as mean ± standard deviation or median (interquartile range), depending on data distribution. Change scores (Δ) were calculated as post-treatment minus baseline values. Between-group comparisons were conducted using independent-sample *t*-tests for normally distributed variables and Mann–Whitney U tests for non-normally distributed variables. Effect sizes are reported for all comparisons: Cohen’s d was used for parametric data, while, for non-parametric data, the effect size was calculated using the formula d = √(Z^2^/(N − 1)), where Z is the standardized test statistic and N is the total sample size (N = 36). A *p*-value < 0.05 was considered statistically significant. VAS = visual analog scale; SPADI = Shoulder Pain and Disability Index; ROM = range of motion.

**Table 4:** Pearson correlation matrix for Group 1.

	VAS Initial	VAS Final	SPADI Initial	SPADI Final	Flexion Gain	Extension Gain	ABD Gain	ER Gain	IR Gain
VAS Initial	1	0.602 * p = 0.014	0.625 ** p = 0.010	0.456 *p* = 0.076	−0.024 *p* = 0.930	0.123 *p* = 0.649	−0.008 *p* = 0.977	0.409 *p* = 0.115	−0.207 *p* = 0.442
VAS Final		1	0.271 *p* = 0.310	0.777 ** p < 0.001	−0.036 *p* = 0.895	0.130 *p* = 0.630	−0.113 *p* = 0.676	0.486 *p* = 0.056	−0.022 *p* = 0.934
SPADI Initial			1	0.581 * p = 0.018	0.111 *p* = 0.682	−0.166 *p* = 0.538	0.272 *p* = 0.309	0.386 *p* = 0.139	−0.062 *p* = 0.819
SPADI Final				1	−0.029 *p* = 0.917	−0.187 *p* = 0.488	0.019 *p* = 0.943	0.390 *p* = 0.135	−0.093 *p* = 0.731
Flexion Gain					1	0.389 *p* = 0.136	0.385 *p* = 0.141	0.019 *p* = 0.944	0.608 * p = 0.012
Extension Gain						1	−0.295 *p* = 0.268	−0.129 *p* = 0.634	0.230 *p* = 0.391
Abduction Gain							1	0.413 *p* = 0.112	0.280 *p* = 0.294
External Rotation Gain								1	0.278 *p* = 0.298
Internal Rotation Gain									1

Note: Correlations are two-tailed Pearson coefficients. * Correlation is significant at the 0.05 level (2-tailed). ** Correlation is significant at the 0.01 level (2-tailed). Moderate to strong correlations are considered for |r| ≥ 0.5.

**Table 5:** Pearson correlation matrix for Group 2.

	VAS Initial	VAS Final	SPADI Initial	SPADI Final	Flexion Gain	Extension Gain	ABD Gain	ER Gain	IR Gain
VAS Initial	1	0.762 ** p < 0.001	0.802 ** p < 0.001	0.478 * p = 0.033	0.334 *p* = 0.150	0.356 *p* = 0.123	0.405 *p* = 0.077	−0.021 *p* = 0.929	0.037 *p* = 0.878
VAS Final		1	0.749 ** p < 0.001	0.624 ** p = 0.003	0.430 *p* = 0.059	0.289 *p* = 0.217	0.339 *p* = 0.144	0.351 *p* = 0.129	0.075 *p* = 0.752
SPADI Initial			1	0.810 ** p < 0.001	0.294 *p* = 0.208	0.218 *p* = 0.357	0.319 *p* = 0.170	0.159 *p* = 0.504	0.139 *p* = 0.559
SPADI Final				1	0.093 *p* = 0.697	0.023 *p* = 0.924	0.045 *p* = 0.851	0.368 *p* = 0.111	−0.117 *p* = 0.622
Flexion Gain					1	0.803 ** p < 0.001	0.822 ** p < 0.001	0.145 *p* = 0.542	0.364 *p* = 0.114
Extension Gain						1	0.809 ** p < 0.001	0.080 *p* = 0.737	0.213 *p* = 0.368
Abduction Gain							1	0.070 *p* = 0.770	0.409 *p* = 0.074
External Rotation Gain								1	0.085 *p* = 0.723
Internal Rotation Gain									1

Note: Correlations are two-tailed Pearson coefficients. * Correlation is significant at the 0.05 level (2-tailed). ** Correlation is significant at the 0.01 level (2-tailed). Moderate to strong correlations are considered for |r| ≥ 0.5.

## Data Availability

The corresponding authors can provide access to the data contained in this study upon request.
